# “T-cell prolymphocytic leukemia (T-PLL), a heterogeneous disease exemplified by two cases and the important role of cytogenetics: a multidisciplinary approach”

**DOI:** 10.1186/2162-3619-1-21

**Published:** 2012-08-20

**Authors:** Carlos A Tirado, Phillip Starshak, Paul Delgado, Nagesh Rao

**Affiliations:** 1 Department of Pathology & Laboratory Medicine, David Geffen UCLA School of Medicine, Los Angeles, CA, 90095, USA; 2 Department of Ecology and Evolutionary Biology, UCLA, Los Angeles, CA, 90095, USA

**Keywords:** T-PLL, Cytogenetics, FISH

## Abstract

T-cell prolymphocytic leukemia (T-PLL) is a rare form of leukemia composed of mature T-cells that usually presents in older people with a median age of 65. Most cases of T-PLL will harbor chromosomal abnormalities involving 14q11.2 (TCR alpha/delta), 14q32 (TCL1) or Xq28 (MTCP-1), abnormalities of chromosome 8, 12p and deletions of the long arm of chromosomes 5, 6, 11 and 13. Cytogenetics, FISH, comparative genomic hybridization (CGH) , SNP arrays with high resolution analysis have provided more precisely frequent submicroscopic gene and genomic lesions as well as breakpoints involved in the pathogenesis of this disease. One of the cornerstones to diagnose T-PLL are cytogenetic analysis. Here we summarize the current cytogenetic findings and we also describe two distinct cases of T-PLL where cytogenetics, FISH , morphologic analysis and flow cytometry helped to diagnose them accurately.

## Introduction

T-cell prolymphocytic leukemia (T-PLL) is a rare mature T-cell lymphoproliferative disorder, which suppresses the immune system through multifactorial processes, thus predisposing the affected patient to a variety of infections, and possibly death [1-3]. Common cytogenetic abnormalities in T-PLL usually include 14q11.2, chromosome 8 rearrangements, 11q abnormalities leading to the deletion of the ATM and MLL genes, and abnormalities on 12p, 5q, 6q and 13q [3-8]. The presence of a combination of these fairly unique structural genetic abnormalities makes chromosome analysis very crucial and extremely helpful to get an accurate and definitive diagnosis of T-PLL as it supplements the immunophenotypic and morphological data. Herein, we present two distinct and diagnostic challenging cases of T-PLL that emphasize the heterogeneity of this disease but showed cytogenetic aberrations that helped to confirm the diagnosis of T-PLL. In addition, we stress the importance of a multidisciplinary approach using the morphologic analysis, flow cytometry, cytogenetic and FISH to accurately diagnose this disease.

### Clinical presentation

**Figure 1:**
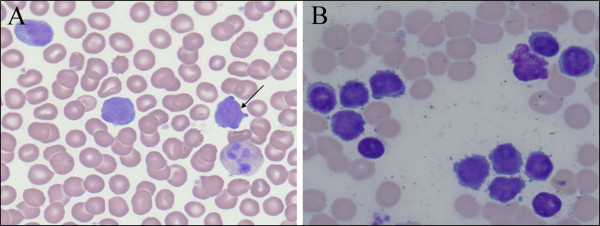
**A: Peripheral blood smear with lymphocytes, note cytoplasmic blebs. B:** Bone marrow aspirate with numerous lymphocytes, note prominent nucleoli.

**Figure 2:**
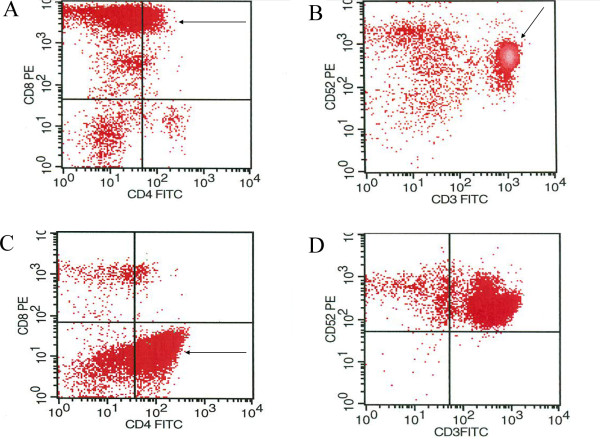
**A: Scatter-plot showing predominant population of T-cells with bright CD8 expression (arrow) B (density-plot): uniform CD52 expression. C**: Scatter-plot showing predominant population of T-cells with dim CD4 expression (arrow) **D**: And heterogeneous CD52 expression.

CASE #1 is an 84 year old male who presented for routine check-up and was found to have an elevated white cell count (WBC) of 22.3×10E3/uL. Peripheral blood (PB) smear showed lymphocytosis that appeared small to intermediate in size with mildly abundant cytoplasm, round to ovoid nuclear contours, and inconspicuous nucleoli. Some showed cytoplasmic blebs (Figure 1A). Flow cytometry of the PB revealed that these atypical lymphocytes were of mature phenotype lacking TdT expression and were positive for alpha-beta T-cell receptor. In addition, the neoplastic T-cells showed bright expression of CD8 and moderate uniform expression of CD52 but no significant loss of pan T-cell antigens was noted (Figures 2A, 2C).

A bone marrow (BM) biopsy revealed an inconspicuous infiltrate of atypical lymphocytes that were highlighted by a *TCL1* immunostain and a diagnosis of 10% involvement of the marrow by atypical CD8 positive mature T-cell lymphocytes was made.

CASE #2 is a 52 year old female with complaints of cough and congestion. A physical exam was notable for tachypnia. Her WBC was 189×10E3/uL. PB smear revealed numerous immature appearing lymphocytes that were intermediate to large in size, had moderate amount of cytoplasm, round nuclear contours, open chromatin, and prominent centrally placed nucleoli consistent with prolymphocytes.

The BM aspirate showed numerous immature appearing lymphocytes with centrally placed nucleolus reminiscent of prolymphocytes (Figure 1B). In addition, flow cytometry showed that the neoplastic cells were mature T-cells as they lacked expression of CD34, HLA-DR, and TdT but were positive for alpha-beta T-cell receptor. These neoplastic T-cells also were positive for CD4 and showed moderate expression of CD52 (Figures 2B, 2D). A BM biopsy revealed a conspicuous infiltrate of large atypical lymphocytes with prominent nucleolus in a background of mutlilineage hematopoiesis and a TCL1 immunostain showed that these atypical lymphocytes were strongly positive.

### Cytogenetics studies

Cytogenetic studies of the bone marrow was performed using conventional cytogenetic techniques. The karyotypes were described according to the ISCN 2009 nomenclature [9].

### CASE #1

**Figure 3:**
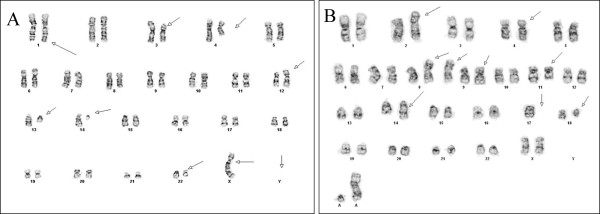
**A: Karyotype 45,t(X;14)(q28;q11.2),-Y,t(1;12;4)(q42;p13;q31.3), add(3)(p13),del(13)(q14q34),del(22)(q12q13)(12)/46,XY(8).B**: Karyotype 48,XX,add(2)(p13),add(4)(p14),i(8)(q10), del(11)(q23),add(14)(q11.2),-17,del(18)(q11.2),+2 ~ 4mar(cp10).

An abnormal clone was seen in 12/20 with loss of the Y chromosome, a (X;14) translocation, a three-way translocation between chromosomes 1q, 12p and 4q, unbalanced rearrangement of 3p, deletions of 13q and 22q. The ISCN karyogram was: 45,t(X;14)(q28;q11.2),-Y,t(1;12;4)(q42;p13;q31.3),add(3)(p13), del(13)(q14q34), del(22)(q12q13)(12)/46,XY(8) (Figure 3A).

### CASE #2

An abnormal clonal population with numerical and structural abnormalities including an isochromosome 8q, structural abnormalities of 2p, 4p, 14q and 18q, deletion 11q, monosomy 17, and up to four marker chromosomes of unknown origin. The ISCN karyogram was: 48,XX,add(2)(p13),add(4)(p14),i(8)(q10),del(11)(q23),add(14)(q11.2),-17,del(18)(q11.2),+2 ~ 4mar (cp10) (Figure 3B).

### Fish studies

FISH analysis was performed using the following probes from Abbott Molecular (Des Plaines, Illinois 60018): The Vysis LSI IGH/MYC/CEP 8 Tri-Color Dual Fusion Probes, the Vysis LSI MYC Dual Color Break Apart Rearrangement Probe, the Vysis TRA/D Break Apart Rearrangement probe, the Vysis CLL FISH Probe Kit and the Vysis LSI BCR/ABL Dual color, Dual Fusion Translocation probe.

### CASE #1

**Figure 4:**
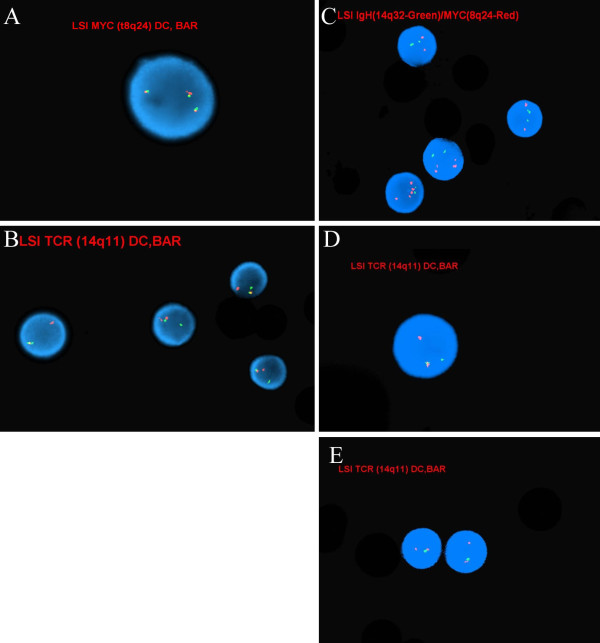
**A: FISH dual-color break apart probe for the C-MYC gene showing three intact copies B: FISH dual-color break apart probe for T-cell receptor alpha-delta showing a split signal suggesting a rearrangement of TCRA/D.C**: FISH showing multiple copies the C-MYC gene (red color). **D**: FISH dual-color break apart probe for T-cell receptor alpha-delta showing a split signal suggesting a rearrangement of TCRA/D in 90% of the cells **E**: 27% of these abnormal cells also showed a deletion of the 3′ TRCA/D.

An abnormal pattern suggestive of a *TCRA/D* gene rearrangement was seen in 96/300 (32.0%) of the cells examined by FISH and 3 copies of 8q24 (MYC) in 19/300 (6.3%) indicating trisomy 8q. The ISCN nomenclature was described as: nuc ish(MYCx3)(19/300), (ATM,TP53x2)(300),(TRA/Dx2)(5′TRA/D sep 3′TRA/Dx1)(96/300) (Figure 4A, 4B).

### CASE #2

An abnormal signal pattern with 3–7 signals for 8q24 in 84.0% (254/300) and 3 copies of chromosome 22q in 8.3% (25/300) of the nuclei examined. In addition, these studies also detected evidence of *TCRA/D* gene rearrangement in 90.0% (270/300) of the nuclei examined and a deletion of the 3′ *TCRA/D* in 26.6% (80/300) of the cells examined by FISH. The ISCN nomenclature was described as: nuc ish (MYCx3 ~ 7,IGH@)*x*2(252/300), (ABL1x2, BCRx3)(25/300),(MLLx2)(300), (ATMx2)(300),(TCRA/Dx2)(5′TCRA/D sep 3′TCRA/Dx1)(190/300)/(5′TCRA/Dx2, 3′TCRA/Dx1)(5′TCRA/D con 3′TCRA/Dx1)(80/300), (TP53x2)(300) (Figure 4C, 4D, 4E).

## Discussion

The two cases seen in the present study demonstrate the immunophenotypic heterogeneity in T-PLL as in case #1 the neoplastic T-cells were CD8 positive while in case#2 the neoplastic T-cells were CD4 positive. In fact, T-PLL can also be double positive for CD4/CD8 or double negative for CD4/CD8. Furthermore, although the expression of CD52 is consistently expressed in T-PLL, and therefore this leukemia is a prime target for campath (aletuzumab) therapy, this feature is not unique to T-PLL as other T-cell lymphoma/leukemias as well as non-neoplastic T-cells are often positive for CD52.

From a morphologic stand-point, the two cases were also quite different with case #1 showing a more mature small lymphocytic appearance with inconspicuous nucleoli whereas case#2 showed larger lymphocytes with prominently placed central nucleolus (prolymphocytes). In fact, it is recognized that a subset of T-PLL cases will have a small lymphocytic appearance and these cases are often referred to as the small cell variant of T-PLL [2,5]. Finally, the clinical scenarios were quite different in both cases with case#1 having a more indolent presentation whereas case#2 had a more typical aggressive presentation.

Complex karyotypes with structural abnormalities [1-8,10-13] including 14q11.2 rearrangements as seen in our cases is observed in 80% of T-PLL cases. These abnormalities include inv(14)(q11.2q32) or the t(14;14)(q11;q32) [3]. Rarely, the t(X;14)(q28;q11) or t(Y;14)(q12;q11) [1]. The 14q32.1 region is very important because it is the locus of the *TCL1* gene and any rearrangement at this locus is sufficient to activate *TCL1* gene expression through juxtaposition of *TCRA* sequences [4,12]. *TCR* stimulation leads to rapid recruitment of *TCL1*, *AKT* and tyrosine kinases to membrane associated activation complexes [4]. However, *TCL1* can also be activated by hypomethylation or loss of methylation on one allele of the TCL1 promoter region [7,12]. Supporting this is the fact that the TCL-1 oncoprotein has been found to be expressed in approximately 70% of T-PLL cases [2]. A study, using SNP-array and gene expression array technologies, 734 genes were found to be directly linked to inv(14), which were differentially expressed and involved in leukemia, cell cycle regulation, apoptosis and DNA repair [3].

Chromosome 8 anomalies as seen in our case are also common in T-PLL. Maljaei et al. [6] and Olivera et al. [1] suggest in their study the synergistic effect as a result of the increased expression of the *MYC* gene (8q24) due to an i(8q) and a deleterious loss of TSG on 8p such as MTUS1 when 8p is deleted in the pathogenesis of T-PLL.

Other findings in T-PLL that may be overlooked by conventional cytogenetics are anomalies of 11q [13] like in case 1 and abnormalities of chromosome 22 like in case 2. Both abnormalities were also confirmed by CGH [10] and SNP-array studies [3]. The minimal deleted regions on chromosome 22 include the genes S*MARCB1* (which encodes the *SWI/SNF* related proteins which might be involved in chromatin remodeling, chromosomal stability, and checkpoint control) and *CHECK*, which seem to be inactivated by biallelic mutation during T-PLL progression. This supports the hypothesis that there might be additional tumor suppressor genes (TSG) on chromosome 22 spanning from the centromere to the 22q12.1 region [11].

CGH studies [10] suggests that there are multiple additional chromosomal abnormalities involved in T-PLL that were previously under recognized by conventional karyotype and FISH analysis including gain of genetic material in many different chromosomal regions including 5p (62%), and 14q (37%), 6p and 21 (both 25%) and loss of genetic material on 13q (37%), 6q, 7q, 16q, 17p, and 17q (25%).

Durig et al. [3] using SNP-based array technique also found deletions in 6p, 8p, 11p, 11q, 12p, 13q and gains in 4q, 5p, 6p and 8q and 14q. Nowak et al. [4] found homozygous deletions on chromosomes 14 and 7 in the T-cell receptor loci, 12p, 5p; heterozygous deletions involving 13q, 17p and 17q and abnormalities involving 5q, 6p, 12p, 13q and 17p.

In conclusion, with the textbook cytogenetic abnormalities revealed in both of these cases that are highly associated with T-PLL we were able to come to a correct diagnosis. Of special note case#2 was initially incorrectly diagnosed as T-lymphoblastic leukemia highlighting that these cases can be diagnostic challenging. Morphologic evaluation, clinical data and techniques with higher resolution such as CGH and SNP-based genomic mapping will elucidate common deletions and gains of chromosomal material unique to T-PLL. Further studies using gene expression profiling techniques are needed to better understand and open more avenues for treatment targets of this disease.

## Competing interests

The authors declare that they have no competing interests.

## Authors’ contributions

CAT lead the whole manuscript writing. PD wrote the first draft and look for literature. PS contribute with discussion and review the manuscript. NR revised everything. All authors read and approved the final manuscript.
